# Two protocols for the detection of oleaginous bacteria using Oil Red O

**DOI:** 10.1007/s00253-024-13177-4

**Published:** 2024-06-15

**Authors:** Roxana A. Silva, Martín A. Hernández, Rainer Kalscheuer, Alexander Steinbüchel, Héctor M. Alvarez

**Affiliations:** 1 Instituto de Biociencias de la Patagonia (INBIOP), Universidad Nacional de la Patagonia San Juan Bosco y CONICET, Km 4-Ciudad Universitaria 9000, Comodoro Rivadavia, Chubut, Argentina; 2 Institute of Pharmaceutical Biology and Biotechnology, Heinrich Heine University, Universitätsstr. 1, 40225 Düsseldorf, Germany; 3 Institut für Molekulare Mikrobiologie und Biotechnologie, Westfälische Wilhelms-Universität, Münster, Germany

**Keywords:** Oil Red O, Triacylglycerols, Oleaginous bacteria, Neutral lipids, Staining protocols

## Abstract

**Abstract:**

The selection of oleaginous bacteria, potentially applicable to biotechnological approaches, is usually carried out by different expensive and time-consuming techniques. In this study, we used Oil Red O (ORO) as an useful dye for staining of neutral lipids (triacylglycerols and wax esters) on thin-layer chromatography plates. ORO could detect minimal quantities of both compounds (detection limit, 0.0025 mg of tripalmitin or 0.005 mg of cetylpalmitate). In addition, we developed a specific, rapid, and inexpensive screening methodology to detect triacylglycerol-accumulating microorganisms grown on the agar plate. This staining methodology detected 9/13 strains with a triacylglycerol content higher than 20% by cellular dry weight. ORO did not stain polyhydroxyalkanoates-producing bacteria. The four oleaginous strains not detected by this screening methodology exhibited a mucoid morphology of their colonies. Apparently, an extracellular polymeric substance produced by these strains hampered the entry of the lipophilic dye into cells. The utilization of the developed screening methodology would allow selecting of oleaginous bacteria in a simpler and faster way than techniques usually used nowadays, based on unspecific staining protocols and spectrophotometric or chromatographic methods. Furthermore, the use of ORO as a staining reagent would easily characterize the neutral lipids accumulated by microorganisms as reserve compounds.

**Key points:**

*• Oil Red O staining is specific for triacylglycerols*

*• Oil Red O staining is useful to detect oleaginous bacteria*

*• Fast and inexpensive staining to isolate oleaginous bacteria from the environment*

## Introduction

The current global energy crisis has forced humans to seek new renewable energies. Production of biodiesel from oleaginous organisms like plants has shown several disadvantages, e.g., competition with human food industry, necessity of lands able to be arable or high amounts of water. In the last years, research in biodiesel production from microorganisms, such as yeasts, fungi, microalgae, and bacteria, has continuously increased (Koreti et al. 2022; Patel et al. 2020).

Some advantages of using bacterial strains in biodiesel production could be the following: (1) conversion of organic waste materials into useful renewable resources; (2) growth of bacterial cells in biorreactors, using less space than it is required for growth of oil seeds; (3) microbial oil does not compete with food for human and animals; (4) biodiesel fuels produced by actinomycetes strains shows an optimal fatty acid composition (only mono-unsaturated fatty acids) and good physical properties (Wahlen et al. 2013); (5) composition of fatty acids can be modified changing culturing conditions, or by genetic and metabolic engineering approaches.

Bacteria are able to accumulate three different reserve lipid compounds, polyhydroxyalkanoic acids (PHA) (Anderson and Dawes 1990), wax esters (WE), and triacylglycerols (TAG) (Alvarez and Steinbüchel 2002). Both TAG and WE can be chemically transesterificated to produce fatty acid esters (biodiesel). Only a small group of bacteria belonging to Gram-positive actinomycetes (mainly *Mycobacterium*, *Rhodococcus*, *Gordonia*) synthesize TAG in large amounts (Alvarez and Steinbüchel 2002). For example, *Rhodococcus opacus* PD630 accumulated almost 80% of TAG per cellular dry weight (CDW) when olive oil was used as the only carbon source (Alvarez et al. 1996). Although a few genera of Gram-negative bacteria synthesizes TAG, their main reserve compound are WE. However, quantities of WE synthesized are lower than TAG concentrations reported in Gram-positive strains, e.g., *Acinetobacter* sp. strain M-1 accumulated only 17% WE (CDW) from *n*-alkanes (Ishige et al. 2002). Therefore, isolation of new oleaginous strains (with the ability to accumulate more than 20% of TAG by CDW) is of great interest for developments of new biotechnological processes.

Conventional methods to detect oleaginous microorganisms are usually time-consuming. They require a separately and differential processing of the samples, using specific equipments or reagents and trained technicians (such as chromatographic methods, colorimetric, or fluorescent quali- or quantification) (Arcos-Hernández et al. 2010; Kimura et al. 2004; Patel et al. 2019a; Wawrik and Harriman 2010).

In prokaryotes, some lipophilic dyes have been utilized to qualify or quantify lipidic compounds, focusing on PHA accumulation: Malachite green (Spiekermann et al. 1999; Sun et al. 1994), tetrazolium violet (Spiekermann et al. 1999), Nile red (Kranz et al. 1997; Spiekermann et al. 1999), Nile blue A (Ostle and Holt, 1982; Spiekermann et al. 1999), and Sudan Black B (He et al. 2010; Ostle and Holt 1982; Schlegel et al. 1970; Spiekermann et al. 1999). These dyes could be directly included in the agar plates or be added on the top of them after growth of the strains, or they were used to stain microscopic cell preparations (Schlegel et al. 1970; Kranz et al. 1997; Spiekermann et al. 1999). In general, Malachite green, tetrazolium violet, and Sudan Black B dyes are unspecific, and they can not discriminate between PHA, TAG, and WE. Conversely, the viable-colony staining method using Nile red and Nile blue A was specially applicable to Gram-negative bacteria with the ability to accumulate PHA, but less suitable to Gram-positive bacteria (Spiekermann et al. 1999).

On the other hand, the main dyes utilized to stain intracellular lipids in eukaryotic cells were Nile red (Fowler and Greenspan 1985; Greenspan et al. 1985) and Oil Red O (ORO) (Fowler and Greenspan 1985; Kimura et al. 2004; Koopman et al. 2001; Ramírez-Zacarías et al. 1992; O’Rourke et al. 2009; Shin et al. 2011; Marquez and Beccaria 2020). Both compounds can be used to qualify or quantify TAG and cholesteryl esters; Nile red could also detect deposits of unesterified cholesterol (Fowler and Greenspan 1985; Ramírez-Zacarías et al. 1992). In this context, Marquez and Beccaria (2020) reported an efficient method based on the use of ORO for both, bioprospecting of oleaginous microalgae and routine analysis of lipid levels in those microorganisms. Microalgae are able to accumulate TAG, but not PHA and wax esters as occur in prokaryotic organisms. ORO was used previously in bacteria to stain lipoid inclusions with unidentified lipids in a strain of *M. smegmatis* (Gale and McLain 1963).

On the other hand, fluorophores such as BODIPY 493/503 or 515/515, LD540 (4,4-difluoro-2.3,5.6-bis-tetramethylene-4-bora-3a,4a-diazo-*s*-indacene), or LipidTOX™ Green have been used to investigate the biosynthesis of lipid droplets in cells using epifluorescent, confocal, two-photon microscopy, and flow cytometry techniques (Grandl and Schmitz 2010; Kacmar et al. 2006; Patel et al. 2019a, b; Spandl et al. 2009).

In this study, we report a staining method for screening of oleaginous bacteria using the dye ORO. This qualitative method is useful for bioprospecting of environmental bacteria with the ability to accumulate high amounts of TAG. In addition, we report a protocol to stain bacterial neutral lipid compounds after thin-layer chromatographic analysis to detect the production of TAG and/or wax esters.

## Materials and methods

### Bacterial strains, media, and chemical compounds

Bacterial strains used in this study are listed in Table 1. Strains were grown in solid mineral salts medium (1.4% agar-agar, w/v) containing 0.1 g/L ammonium chloride (MSM0.1; nitrogen deficient condition) (Schlegel et al. 1961). Sodium gluconate or glycerol (1 or 0.3% w/v, respectively) was added as sole carbon source. Master plates of nutrient broth (NB) (0.8%, w/v) were prepared.

**Table 1:** Bacterial strains and lipid compounds accumulated in the conditions used in this study

Bacterial strains	Lipidic compound*	References
*Acinetobacter baylyi* ADP1	WE	Juni and Janik (1969)
*Bacillus megaterium* PV447	PHA	Tao and Vary (1991)
*Cobetia* sp. PC412	PHA/TAG	Sediment isolate
*Escherichia coli* ATCC 25922	nd	
*Mycobacterium ratisbonense* SD4	PHA/TAG	Berekaa and Steinbüchel (2000)
*Rhodococcus erythropolis* 17	PHA/TAG	Alvarez (2003)
*Rhodococcus erythropolis* DSM43060	PHA/TAG	Brandao et al. (2003)
*Rhodococcus fascians* F7	PHA/TAG	Herrero et al. (2016)
*Rhodococcus jostii* RHA1	TAG	Seto et al. (1995)
*Rhodococcus jostii* 602	PHA/TAG	Silva et al. (2010)
*Rhodococcus jostii* 006	PHA/TAG	Herrero et al. (2016)
*Rhodococcus jostii* G212	PHA/TAG	Herrero et al. (2016)
*Rhodococcus jostii* 016	PHA/TAG	Bourguignon et al. (2014)
*Rhodococcus jostii* 346	PHA/TAG	Herrero et al. (2016)
*Rhodococcus opacus* PD630	TAG	Alvarez et al. (1996)
*Rhodococcus wratislaviensis* V	PHA/TAG	Herrero et al. (2018)
*Rhodococcus* sp. H	PHA/TAG	Soil isolate
*Rhodococcus* sp. 20	PHA/TAG	Alvarez (2003)

Abbreviations: *nd*, not detected; *PHA*, polyhydroxyalkanoates; *TAG*, triacylglycerols; *WE*, wax esters. *Determined by TLC/GC analysis

Oil Red O (C_26_H_24_N_4_O) was obtained from Sigma Aldrich (St. Louis, MO, USA), whereas other chemicals were from Sigma Aldrich, Merck (Darmstadt, Germany), or Fluka Chemika (Buchs, Switzerland).

### Detection of lipid compounds by ORO staining procedures

An ORO working solution was prepared as described by Ramírez-Zacarías et al. (1992), by dissolving 4.2 g of ORO in 1200 mL absolute 2-propanol and leaving overnight without stirring at room temperature. The solution was filtered through a Whatman filter and then diluted with 900 mL of distilled water; this solution was kept overnight at 4 °C without stirring and filtered twice to generate the working solution.

Two protocols were developed using ORO as staining compound. To test the utility of ORO as a staining reagent of neutral lipid compounds, different samples were run (see below) by thin-layer chromatography (TLC). After running, the plates were submerged in the ORO working solution and incubated at 37 °C for 10 min; then, they were washed in 2-propanol 60% (v/v) for 20 min. To determine sensibility of ORO under these conditions, different concentrations (mg) of tripalmitin and cetylpalmitate (between 0.0025 and 0.09 mg) were analyzed by TLC analysis and stained as described previously.

To determine the usefulness of ORO to screen for TAG-accumulating bacteria, different strains were grown in MSM0.1 agar plates with an appropriate carbon source at 28 °C for 72 h (time needed to accumulate high levels of TAG in *R. opacus* PD630). More than 50 bacterial strains can be grown as groove in the same agar plate. Parallelly, master plates on NB were prepared. Then, MSM0.1 agar plates were covered with the working solution and incubated at three temperatures (20, 28, and 37 °C) and times of exposure (30, 60, and 120 min). After slowly removing the working solution by pouring, the Petri dishes were washed with 40% (v/v) ethanol for 1 min to remove the excess of dye and colonies were macroscopically observed. *R. opacus* PD630 and *A. baylyi* ADP1 were used as TAG- and WE-accumulating controls, respectively, while *Bacillus megaterium* PV447 (PHA-accumulating strain) and *Escherichia coli* ATCC25922 (which does not synthesize any reserve lipid compound) were used as negative neutral lipid controls. Due to the exposure to the ORO solution and ethanol causes cell death, viable cells of selected strains must be recovered from NB master plates.

### Lipid content according to chromatographic analysis

Cells grown on MSM0.1 agar plates for 72 h were harvested, washed once with sterile NaCl solution (0.85%, w/v), and dried at 37 °C. To analyze lipid accumulation, 5–10 mg of dried cells were extracted with methanol-chloroform (MeOH:CHCl_3_, 1:2, v/v). An aliquot of each whole cells extract was analyzed by TLC on pre-coated 60-silica gel plates (ALUGRAM® Xtra SIL G, Macherey-Nagel, Düren, Germany) applying *n*-hexane-diethyl ether-acetic acid (80:20:1, v/v/v) as solvent system. Lipid fractions were visualized using either iodine vapor or ORO solution as described above. Tripalmitin and cetylpalmitate (Merck, Darmstadt, Germany) were used as standards.

To determine the fatty acid contents, 3–10 mg dried cells were subjected to methanolysis in the presence of equal parts of CHCl_3_ and MeOH: H_2_SO_4_ solution (85:15, v/v), at 100 °C for 3 h. The resulting fatty acid methyl esters (0.2 µL of each one) were analyzed by gas chromatography (GC) in a HP5890A gas chromatograph equipped with an InnoWAX capillary column (30 m × 0.52 mm × 1 μm) and a flame ionization detector. Helium (13 mm/min) was used as carrier gas. The temperatures of the injector and detector were 270 °C and 320 °C, respectively. The efficient separation of the methyl esters was obtained using the following program: the oven temperature was maintained at 90 °C for 5 min, then increased at a rate of 6 °C/min up to 240 °C, and held at 240 °C for 17 min. For quantitative analysis, tridecanoic acid was used as internal standard.

### Taxonomic characterization

Total DNA was extracted by the method of Chachaty and Saulnier (2000). Amplification reactions were performed using a thermal cycler (Eppendorf Mastercycler Personal) and universal primers plus 0.2-1 U of Taq DNA polymerase (Inbio Highway, Argentina). The identity of each strain was obtained by comparison of the 16S rDNA sequences with the GenBank database using the BLAST tool (Altschul et al. 1990).

## Results

In this work, we developed two simple methodologies using the dye ORO, which allow the detection of neutral lipid-accumulating bacteria, preferentially strains with a high content of TAG.

**Fig. 1:**
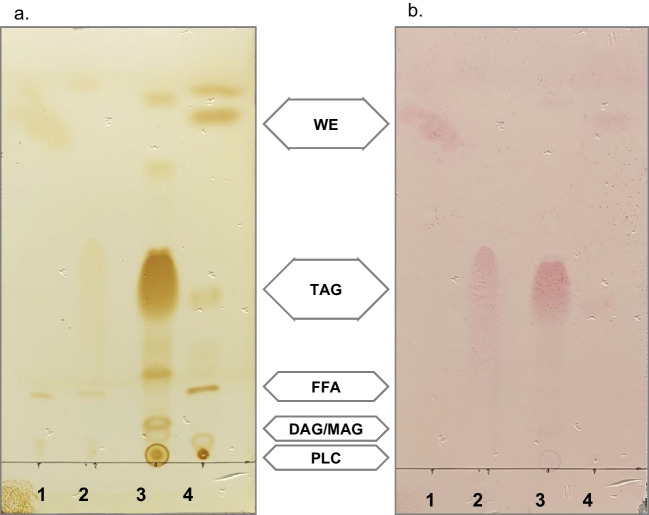
TLC plates of cellular lipid extracts revealed with **a** iodine vapors and **b** ORO solution. References: 1, cetylpalmitate; 2, tripalmitin; 3, *R. opacus* PD630; 4, *A. baylyi* ADP1. WE, wax esters; TAG, triacylglycerols; FFA, free fatty acids; DAG, diacylglycerols; MAG, monoacylglycerols; PLC, polar lipid compounds

**Fig. 2:**
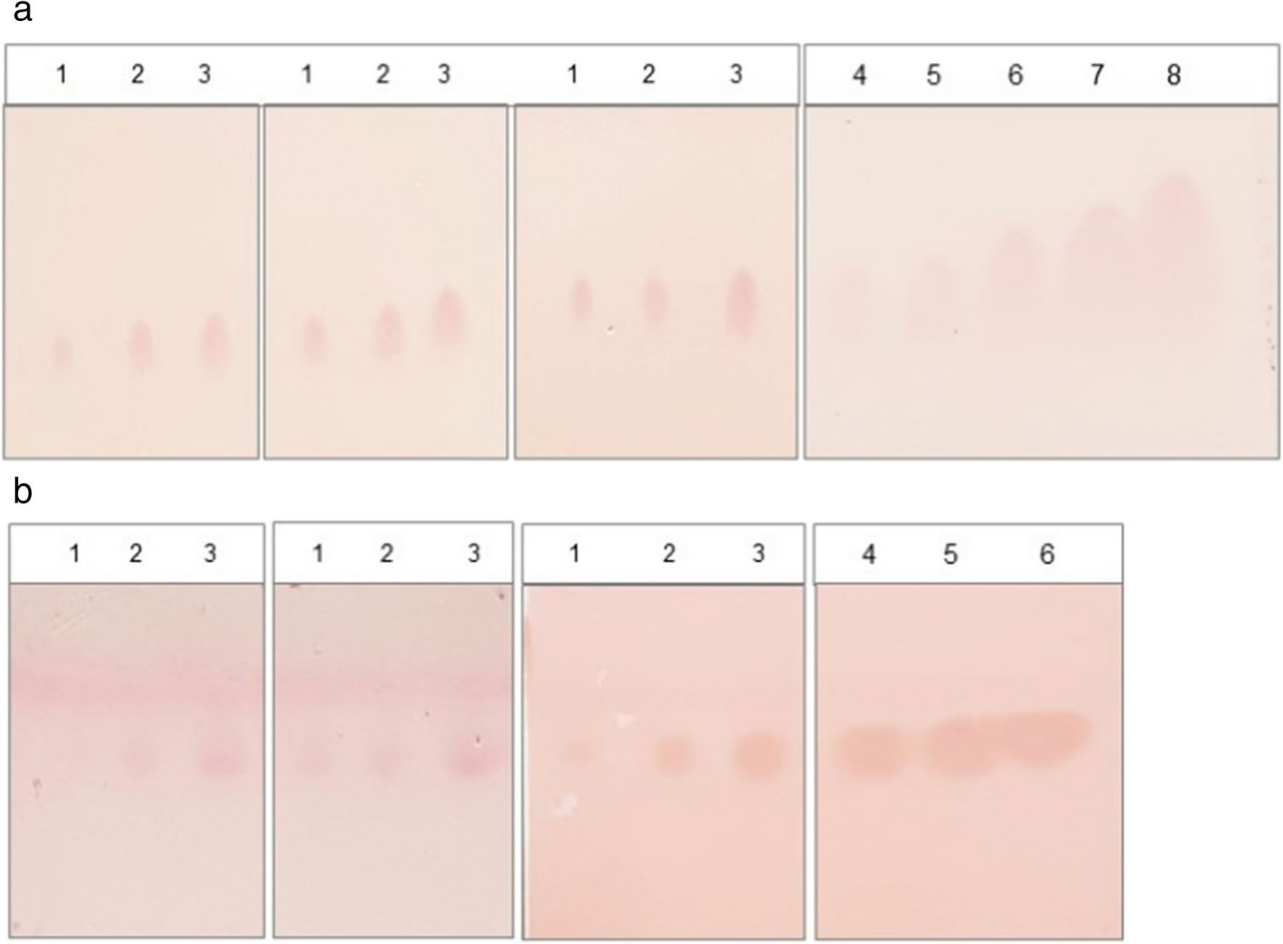
ORO-stained TLC plates with different concentrations of TAG and wax ester standards, respectively. **a** Triacylglycerols (mg): (1) 0.0025, (2) 0.005, (3) 0.01, (4) 0.02, (5) 0.03, (6) 0.05, (7) 0.07, and (8) 0.09. **b** Wax esters (mg): (1) 0.005, (2) 0.01, (3) 0.02, (4) 0.03, (5) 0.04: (6) 0.05. Determinations of the three samples with the lower concentrations of TAG and wax esters (1, 2, 3 in the figures), respectively, were performed in triplicate. Samples 1 of TAG and wax ester standards represent the limit of detection, respectively

First, we evaluated the affinity of ORO towards bacterial neutral lipid compounds. Cellular lipid extracts of *R. opacus* PD630 and *A. baylyi* ADP1 (producers of TAG and WE, respectively) (Alvarez et al. 1996; Kalscheuer and Steinbüchel 2003) and TAG/WE standards were analyzed by TLC using a standard solvent system for neutral lipids. After running, TLC plates were soaked with ORO solution as staining reagent. While iodine vapors detected many cellular lipidic compounds (TAG, WE, free fatty acids, diacylglycerols, monoacylglycerols, phospholipids, and others unknown compounds), ORO solution stained with more affinity TAG and WE (Fig. 1). Interestingly, ORO-stained spots in TLC plates were visible after several days after exposition to the stain, whereas iodine staining dissapeared in few minutes. According to a sensitivity assay performed on TLC plates, ORO dye was able to detect up to 0.0025 mg of TAG and 0.005 mg of WE (Fig. 2).

Then, we optimized the conditions for the use of ORO for the screening of oleaginous bacteria. Cells of strains used as controls were grown on agar plates under conditions promoting storage lipid accumulation (nitrogen deficient media). Subsequently, cells were stained with the ORO solution as was described above, for three different times and at different temperatures of exposure. Colonies of *R. opacus* PD630 (TAG-positive control) stained at 28/37 °C for 120 min showed a better coloration than those stained at 20º C or for 30/60 min (data not shown). There were no differences between results observed at 28 or 37 °C. Considering that 28 °C is a common incubating temperature in environmental microbiology laboratories, we selected 28 °C for 120 min as the best condition to obtain well ORO-stained cells. Using these conditions, TAG-positive control colonies developed an intense red coloration, while WE-, PHA-, or negative-control maintained natural coloration of colonies (Table 2).

**Table 2:**
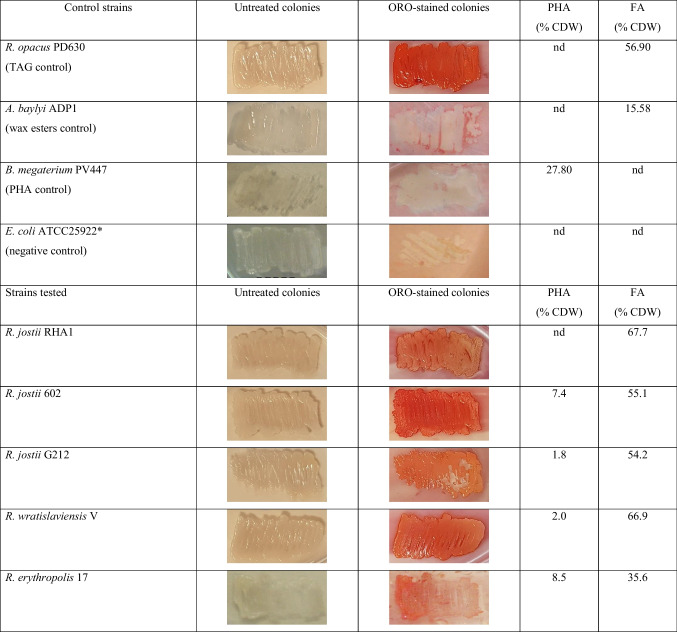

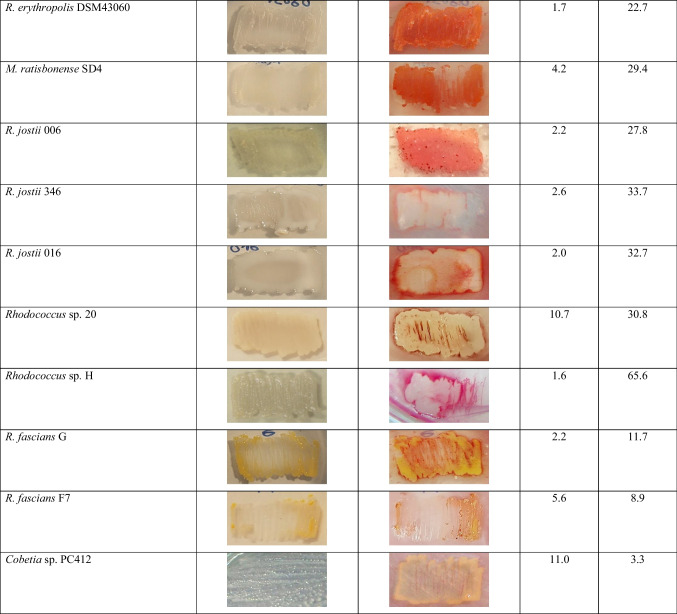
GC results and images of ORO-stained colonies of the strains tested in this study. Cells were cultivated in solid mineral medium with 0.1 g/L NH_4_Cl and an appropriate carbon source at 28 °C for 72 h and stained with ORO solution at 28 °C for 120 min

*This strain is not able to accumulate lipid reserve compounds. Abbreviations: *CDW*, cellular dry weight; *FA*, fatty acids; *nd*, not detected; *ORO*, Oil Red O; *PHA*, polyhydroxyalkanoates; *TAG*, triacylglycerols

Next, we screened 15 other strains of our own collection (including strains isolated from soil or sediment samples from Patagonia, Argentina), using this methodology (Table 1). According to TLC or GC analyses, these strains were able to synthesize TAG or PHA, or both compounds; none strains synthesized WE under the nitrogen deficient conditions used (Table 2). Twelve of these strains accumulated more than 20% CDW of intracellular TAG, while *Cobetia* sp. PC412 accumulated 11% CDW of PHA (Table 2). When screening all strains with ORO solution, 9 of 13 TAG-accumulating strains were detected (it did not stain strain PC412); these strains incorporated the dye and changed their natural coloration to an intense orange or red color (Table 2). The color intensity was not proportional to the amount of TAG stored by each strain (Table 2). However, four TAG-accumulating strains could not be detected by this staining methodology (*Rhodococcus* sp. strains 346, 016, 20, and H) (Table 2). The main difference between these microorganisms to the other nine strains was their ability to synthesize a hydrophilic extracellular polymeric substance (EPS) under the nitrogen deficient conditions used (Table 3).

**Table 3:**
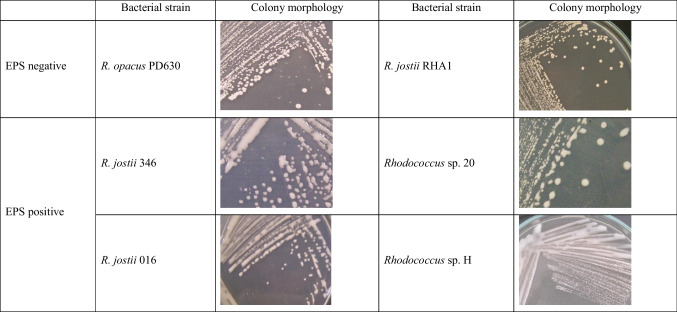
Colony morphologies of six strains studied in this work. Cells were cultivated for 72 h in solid mineral medium with 0.1 g/L NH_4_Cl and gluconate added as carbon source at 1% (w/v)

## Discussion

ORO has been widely used to study the genesis or the conversion of adipocytes (which contain mainly TAG) in eukaryotic organisms (Fowler and Greenspan 1985; Ramírez-Zacarías et al. 1992). Furthermore, in last years, this dye has been used to detect and quantify TAG in lower eukaryotic organisms such as fungi, yeast, microalgae, or nematods (O’Rourke et al. 2009; Shin et al. 2011; Marquez and Beccaria 2020). Shin et al. (2007) patented an ORO staining methodology applicable to fungi and yeast fixed cells. The goal of this method is to allow a fast microscopic analysis of accumulation and metabolism of TAG, without grinding or deforming of cells. Besides, Shin and co-workers proposed the utility of their methodology for bacteria.

In this work, we used conventional ORO solution (Ramírez-Zacarías et al. 1992) in two methodologies to detect TAG-accumulating bacterial strains: as a staining reagent of neutral lipids separated by TLC or as the dye employed in a colony staining method allowing large-scale screening approaches. Our results showed that ORO is able to stain both neutral lipid compounds accumulated by bacteria (TAG and WE) when they were extracted from the cells. Under the tested conditions, the sensitivity of ORO for TAG in TLC plates was higher in comparison to WE. In this way, ORO staining can be used as an alternative method, qualitative or semi-quantitative to some extent, to reveal the presence of TAG and/or wax esters after TLC separation of neutral lipids extracted from bacterial cells.

On the other hand, we developed a method useful to screen a large number of bacterial strains at the same time, applying ORO solution directly on the top of the agar plates. Nile red and Sudan Black B dyes are used in a similar way, but both dyes are non-specific and they can not discriminate between PHA or neutral lipids (Schlegel et al. 1970; Kranz et al. 1997).

Marquez and Beccaria (2020) used ORO staining for screening oleaginous microalgal cells and quantification of lipids. They adapted an ORO staining protocol to be applied to microalgae in suspension for observing under a light microscope. In addition, the authors developed a method to quantify lipids by measuring the captured ORO with a spectrophotometer working in the visible region of the spectrum. In contrast to that study, our ORO staining protocol is directly applied to bacterial colonies and not to cells in suspensions for microscopic observation. Furthermore, our protocol emphasizes the qualitative aspect of lipid accumulation to select oleaginous bacteria (TAG-producers), but it is not conceived as a quantitative method. Once the oleaginous bacteria have been selected by this method, the strains can be quantitatively analyzed using standard analytical methods, such as gas chromatography analysis. This procedure is particularly important when applied to prokaryotes, since these microorganisms, unlike microalgae, fungi and yeasts, have the ability to produce different types of storage lipids, such as PHA, TAG, and wax esters. The protocol developed in this study allows the screening and detection of oleaginous bacteria in a single step, unlike non-specific lipophilic dyes such as Sudan Black B or Nile red. Its disadvantage is that ORO presence in the culture media does affect growth of bacterial cells, in comparison to Nile blue A and Nile red-staining methods that can be directly included in the medium to estimate the presence of PHA in viable colonies (Spiekermann et al. 1999). In contrast, ORO must be dissolved in an organic solvent and then is poured onto the agar plates. This procedure kills cells due to the toxicity of the solvent used for solubilizing the lipophilic dye. However, this inconvenience can be overcome using master plates that are not treated by the staining procedure to retain viable cells.

Our results showed that this screening methodology allows specifically detecting oleaginous strains (9/13, including TAG-accumulating control strain). Neither PHA- nor WE-producer strains were detected. This is an important result considering all current biotechnological approaches focused on this kind of bacterial lipid compound (Thomson et al. 2010; Koreti et al. 2022; Patel et al. 2020). However, this staining procedure failed to detect four oleaginous bacterial strains, which showed a mucoid morphology. This result could be related to the synthesis of anhydrophilic extracellular polimeric substance (EPS) by these strains under the culture conditions used (nitrogen deficient conditions). In a previous study, we showed that low levels of nitrogen not only promotes synthesis and accumulation of TAG in bacteria, but also induces synthesis of EPS (Alvarez et al. 2004). On the other hand, other studies demonstrated that *Rhodococcus* members are able to produce exopolysaccharides enriched in mannose and glucose as monosaccharide components (Li et al. 2023). Apparently, these substances interfer with the entry of ORO (an hydrophobic compound) into cells, avoiding its complete dissolution in the intracellular TAG-matrix producing an improper staining. Liu et al. (1998) also reported a differential uptake of Sudan Black B by *Rhizobium meliloti* wild type, which synthesizes exopolysaccharides, and its exopolysaccharide-deficient mutant. They found that exopolysaccharide-producing strains of *R. meliloti* excluded the stain Sudan Black B and that exopolysaccharide-deficient mutants readily incorporated this stain (Liu et al. 1998).

In conclusion, this non viable-colony staining method allows specifically the detection of TAG-producing microorganisms, enabling testing of approximately 60 bacterial colonies in the same plate and discriminating strains which accumulate PHA or WE. This simple, inexpensive, and rapid methodology saves time for analysis and laboratory supplies and can be used for selection of oleaginous strains applicable in biotechnological approaches, such as synthesis of bacterial oils to biodiesel production.

## Data Availability

All data supporting the findings of this study are available within the paper.
